# The Urinary Excretion of Uromodulin is Regulated by the Potassium Channel ROMK

**DOI:** 10.1038/s41598-019-55771-x

**Published:** 2019-12-20

**Authors:** Guglielmo Schiano, Bob Glaudemans, Eric Olinger, Nadine Goelz, Michael Müller, Dominique Loffing-Cueni, Georges Deschenes, Johannes Loffing, Olivier Devuyst

**Affiliations:** 1 Institute of Physiology, University of Zurich, Zurich, Switzerland; 2 Institute of Anatomy, University of Zurich, Zurich, Switzerland; 3 Hôpital Robert-Debré, AP-HP, Paris, France

**Keywords:** Nephrons, Nephrology

## Abstract

Uromodulin, the most abundant protein in normal urine, is produced by cells lining the thick ascending limb (TAL) of the loop of Henle. Uromodulin regulates the activity of the potassium channel ROMK in TAL cells. Common variants in *KCNJ1*, the gene encoding ROMK, are associated with urinary levels of uromodulin in population studies. Here, we investigated the functional link between ROMK and uromodulin in *Kcnj1* knock-out mouse models, in primary cultures of mouse TAL (mTAL) cells, and in patients with Bartter syndrome due to *KCNJ1* mutations. Both global and kidney-specific *Kcnj1* knock-out mice showed reduced urinary levels of uromodulin paralleled by increased levels in the kidney, compared to wild-type controls. Pharmacological inhibition and genetic deletion of ROMK in mTAL cells caused a reduction in apical uromodulin excretion, reflected by cellular accumulation. In contrast, NKCC2 inhibition showed no effect on uromodulin processing. Patients with Bartter syndrome type 2 showed reduced urinary uromodulin levels compared to age and gender matched controls. These results demonstrate that ROMK directly regulates processing and release of uromodulin by TAL cells, independently from NKCC2. They support the functional link between transport activity and uromodulin in the TAL, relevant for blood pressure control and urinary concentrating ability.

## Introduction

Uromodulin (previously named Tamm-Horsfall protein) is the most abundant protein excreted in normal human urine. This extracellular matrix-type protein is essentially produced and excreted in the urine by the epithelial cells lining the thick ascending limb (TAL) of Henle’s loop^1^. A sizeable expression of uromodulin, approximately 10% of the level observed in the TAL, is also detected in the early part of the distal convoluted tubule (DCT)^2^. Important roles for uromodulin include protection against urinary tract infections by binding to type 1 – fimbriated uropathogenic *E. coli*^3,4^; reduction of kidney stone formation by binding calcium oxalate crystals^5,6^; and regulation of the Na^+^-cotransporters NKCC2 (SLC12A1) and NCC (SLC12A3) in the apical membrane of the TAL and DCT cells, respectively^2,7,8^. The functional importance of uromodulin is also supported by genetic evidence. Genome-wide association studies (GWAS) have shown that common variants in the promoter of the *UMOD* gene that encodes uromodulin are associated with estimated glomerular filtration rate (eGFR) and the risk of chronic kidney disease (CKD) and hypertension in the general population^9–11^. Furthermore, rare mutations in *UMOD* are causing autosomal dominant tubulointerstitial kidney disease (ADTKD-*UMOD*), a gain of toxic function disorder due to abnormal accumulation of mutant uromodulin aggregates in the TAL, leading to kidney fibrosis and CKD^12,13^.

In contrast with its roles in the kidney, the regulation of uromodulin production and release in the urine remains poorly characterized. Uromodulin is a glycosylphosphatidyl-inositol (GPI)-anchored protein that is targeted to the apical membrane of tubular cells, where it is subsequently cleaved and released in the lumen by the serine protease hepsin^14^. Once cleaved and released in the lumen, the uromodulin monomers assemble via their Zona Pellucida (ZP) domain to form a dense matrix of high-molecular weight polymers constituting hyaline casts^1^. Based on a meta-GWAS of urinary uromodulin level in the general population, a candidate gene analysis revealed that the gene with the strongest association with urinary uromodulin was *KCNJ1* coding for the apical K^+^ channel ROMK^15^.

ROMK co-localizes with uromodulin at the apical membrane of TAL cells, where it recycles part of the reabsorbed K^+^ from the intracellular compartment back to the tubular lumen – a process that is essential for maintaining the activity of NKCC2^16,17^. Inactivating mutations of *KCNJ1* cause antenatal Bartter syndrome type 2, a rare disorder characterized by salt wasting, hypokalemic metabolic alkalosis, hypercalciuria, and secondary nephrocalcinosis^18^. Renigunta *et al*. identified uromodulin as an interaction partner of ROMK in yeast-two-hybrid screens, with transport activity and surface expression of ROMK being increased when co-expressed with uromodulin in *Xenopus laevis* oocytes^19^. Furthermore, *Umod*^−/−^ mice showed defective apical targeting of ROMK, reflecting accumulation of the channel in intracellular vesicles^19^.

The genetic and functional data summarized above suggest a complex interplay between ROMK and uromodulin, which could be relevant *in vivo* – as modifications in TAL function increasingly appear to influence the release of uromodulin in urine^20–22^. In the present study, we used various *Kcnj1* knock-out (KO) mouse models, differentiated primary cell cultures obtained from mouse TAL and urine samples from individuals harbouring inactivating mutations of *KCNJ1* to investigate the effect of ROMK deficiency on uromodulin expression, processing and release in the urine.

## Results

### Uromodulin expression and processing in *Kcnj1* knock-out mice

**Figure 1:**
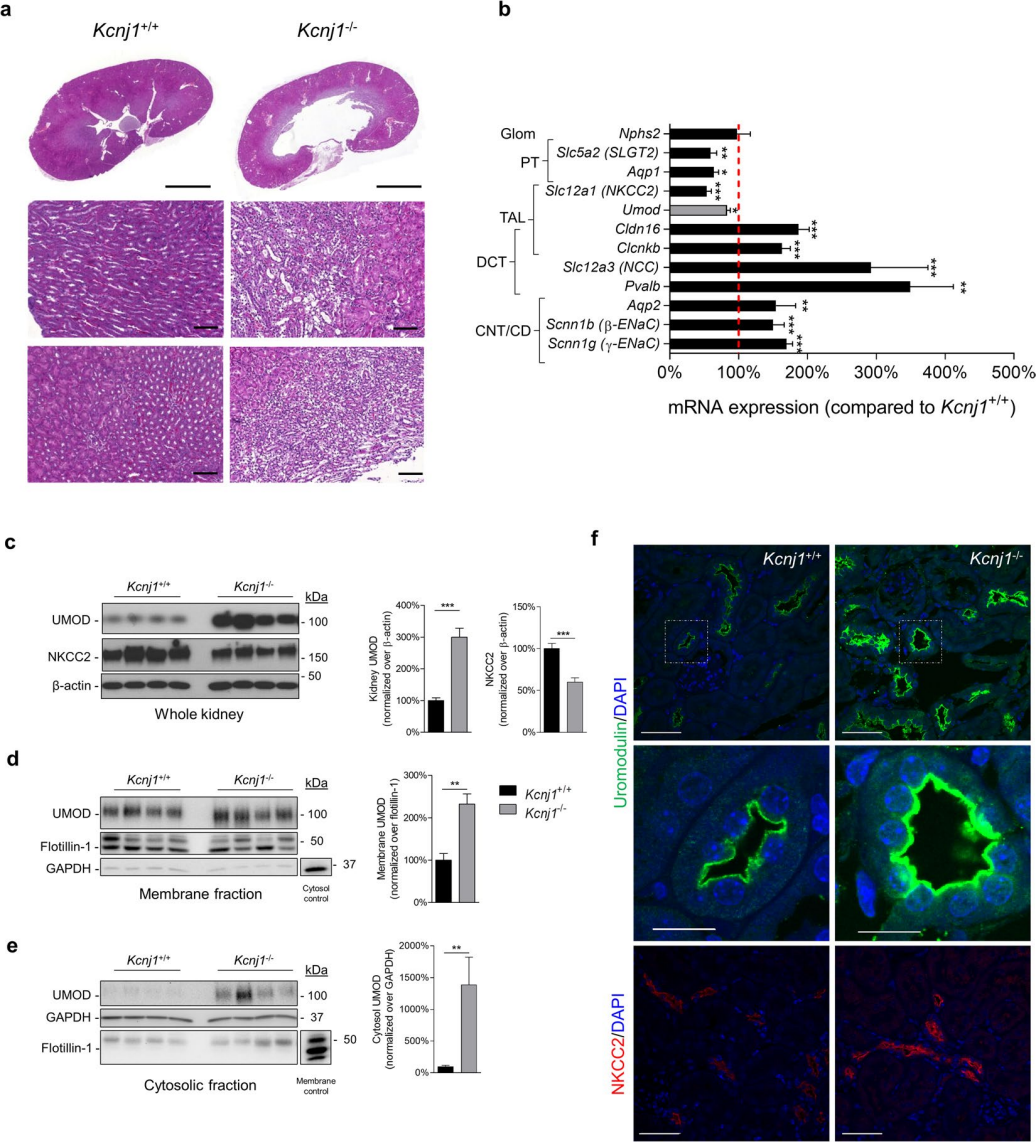
Histological characterization, transcript analysis and uromodulin processing in *Kcnj1*^−/−^ mice. (**a**) Haematoxylin and eosin (H&E) staining of 12 weeks *Kcnj1*^+/+^ and *Kcnj1*^−/−^ kidneys. Scale bars: low magnification - 2 mm, high magnification - 100 μm (**b**) Quantitative PCR analysis of tubular marker genes in *Kcnj1*^−/−^ kidneys. Glom (Glomerulus): *Nphs2* (Podocin); PT (Proximal Tubule): *Slc5a2* - Sodium/glucose cotransporter 2 (SGLT2), *Aqp1* - Aquaporin 1; TAL (Thick Ascending Limb): *Slc12a1* - Na/K/Cl cotransporter 2 (NKCC2), *Umod* - Uromodulin; DCT (Distal Convoluted Tubule): *Slc12a3* - Na/Cl cotransporter (NCC), *Pvalb* - Parvalbumin; CNT/CD (Connecting Tubule/Collecting Duct): *Aqp2* - Aquaporin 2, *Scnn1b* - Sodium channel epithelial 1 beta subunit (β-ENaC), *Scnn1g* - Sodium channel epithelial 1 gamma subunit (γ-ENaC); *Cldn16* - Claudin 16 and *Clcnkb* (Cl channel Kb) are localized in both TAL and DCT. (**c**) Representative Western blot analysis on whole kidney lysates of 12 weeks *Kcnj1*^+/+^ and *Kcnj1*^−/−^ mice. β-actin was used as a loading control. (**d**) Representative Western blot analysis on kidney membrane fractions of 12 weeks *Kcnj1*^+/+^ and *Kcnj1*^−/−^ mice. Flotillin-1 was used as a loading control, whereas GAPDH was used to test cytosolic contamination. (**e**) Representative Western blot analysis on kidney cytosolic fractions of 12 weeks *Kcnj1*^+/+^ and *Kcnj1*^−/−^ mice. GAPDH was used as a loading control, whereas Flotillin-1 was used to test membrane contamination. (**f**) Immunofluorescence analysis for UMOD (green) and NKCC2 (red) in *Kcnj1*^+/+^ and *Kcnj1*^−/−^ serial kidney sections. Scale bars: low magnification - 50 μm, high magnification - 15 μm. Full length blot images can be found in Supplementary Figure S2.

The interplay between ROMK and uromodulin was first investigated in the constitutive, global *Kcnj1* KO compared to control littermates. Only male *Kcnj1*^−/−^ mice were included in this study due to their less severe phenotype and higher survival rate^23^. Histological examination of the *Kcnj1*^−/−^ kidneys evidenced hydronephrosis and severe thinning of the medulla (Fig. 1a). At the mRNA level (Fig. 1b), global ROMK deficiency was reflected by a slight downregulation of uromodulin expression (*Umod*: 83 ± 2% of control; *P* < 0.05) in *Kcnj1*^−/−^ compared to *Kcnj1*^+/+^ kidneys, whereas other TAL genes were either significantly decreased (*Slc12a1*: 54 ± 3% of control; *P* < 0.0001) or increased (*Clcnkb*: 163 ± 6% of control; *P* < 0.0001; *Cldn16*: 187 ± 7% of control; *P* < 0.0001). Of note, ROMK deletion was reflected by a significant upregulation of genes expressed in the DCT, including *Slc12a3* (292 ± 37% of control; *P* < 0.001) and *Pvalb* (349 ± 63% of control; *P* < 0.01) and in the connecting tubule/collecting duct (CNT/CD) segments, including *Aqp2* (154 ± 13% of control; *P* < 0.01), *Scnn1b* (150 ± 7% of control; *P* < 0.001) and *Scnn1g* (170 ± 4% of control; *P* < 0.0001), whereas the expression of proximal tubule (PT) genes *Sglt2* (59 ± 4% of control; *P* < 0.01) and *Aqp1* (64 ± 3% of control; *P* < 0.05) was significantly decreased in *Kcnj1*^−/−^ mice (Fig. 1b).

Immunoblotting analyses confirmed that NKCC2 protein levels were significantly lowered in total kidney lysates from *Kcnj1*^−/−^ mice (60 ± 5% of control; n = 5; *P* < 0.001), strongly contrasting with total kidney uromodulin levels, which were significantly higher (300 ± 29% of control; n = 5; *P* < 0.001) in *Kcnj1*^−/−^ kidneys (Fig. 1c). To identify the cellular localization of the increased uromodulin signal, we separated membrane and cytosolic fractions obtained from the kidneys of *Kcnj1*^−/−^ and *Kcnj1*^+/+^ mice. The purity of these fractions was assessed by probing with anti-GAPDH (membrane) and anti-flotillin-1 (cytosolic). Compared to *Kcnj1*^+/+^ kidneys, we detected an increase in uromodulin both in the membrane (232 ± 24% of control, n = 5; *P* < 0.01; Fig. 1d) and cytosolic (1389 ± 433% of control, n = 5; *P* = 0.003; Fig. 1e) fractions from *Kcnj1*^−/−^ kidneys.

The modifications of uromodulin expression were further analysed using confocal microscopy on sections of *Kcnj1* mouse kidneys (Fig. 1f). Although uromodulin showed a similar localization at or close to the apical membrane of TAL cells (sub-apical vesicles) in both genotypes, the signal was more intense in the *Kcnj1*^−/−^ compared to *Kcnj1*^+/+^ kidneys. Of note, the apical staining of NKCC2 was also stronger in *Kcnj1*^−/−^ kidneys, despite the global reduction of NKCC2 expression, suggesting an overall reduction in TAL mass.

Metabolic studies indicated that *Kcnj1*^−/−^ mice presented similar body weight, BUN and creatinine clearance levels than *Kcnj1*^+/+^ littermates, despite polyuria with diluted urine (Table 1). The plasma Na^+^ and Cl^-^ concentrations were similar between *Kcnj1*^−/−^ and *Kcnj1*^+/+^ mice, except for a mild decrease in plasma K^+^ concentration in *Kcnj1*^−/−^ animals.

**Table 1:** Physiological and biochemical parameters in *Kcnj1* mice.

	*Kcnj1*^+/+^	*Kcnj1*^−/−^	*N*
Body weight (g)	28.5 ± 0.7	28.5 ± 0.9	11/11
Urine volume (µL/16 h)	977 ± 134	2829 ± 440***	11/11
Plasma Creatinine (µmol/L)	20 ± 1	27 ± 2	6/6
Blood urea nitrogen (mg/dL)	28 ± 1	31 ± 3	6/6
Plasma [Na^+^](mmol/L)	154 ± 1	153 ± 1	6/6
Plasma [K^+^] (mmol/L)	5.7 ± 0.2	4.9 ± 0.3*	6/6
Plasma [Cl^−^] (mmol/L)	109 ± 0	106 ± 1	6/6
Plasma [Ca^2+^] (mmol/L)	2.59 ± 0.02	2.70 ± 0.05	6/6
Urinary Creatinine (mg/dL)	51.4 ± 4.9	14.9 ± 1.2***	11/11
Urinary osmolality (mOsm/kgH_2_O)	1780 ± 108	645 ± 24***	11/11
Na^+^ excretion (µmol/16 h)	110 ± 16	87 ± 15	11/11
K^+^ excretion (µmol/16 h)	200 ± 35	185 ± 25	11/11
Cl^−^ excretion (µmol/16 h)	143 ± 28	120 ± 20	11/11
Ca^2+^ excretion (µmol/16 h)	1.01 ± 0.25	1.98 ± 0.74	11/5
C_Cr_ (mL/min)	0.16 ± 0.04	0.14 ± 0.01	6/6

Values are presented as average ± SEM. *P ≤ 0.05, ***P ≤ 0.001 *Kcnj1*^+/+^ versus *Kcnj1*^−/−^ mice.

n: number of animals; C_Cr_: Creatinine clearance.

**Figure 2:**
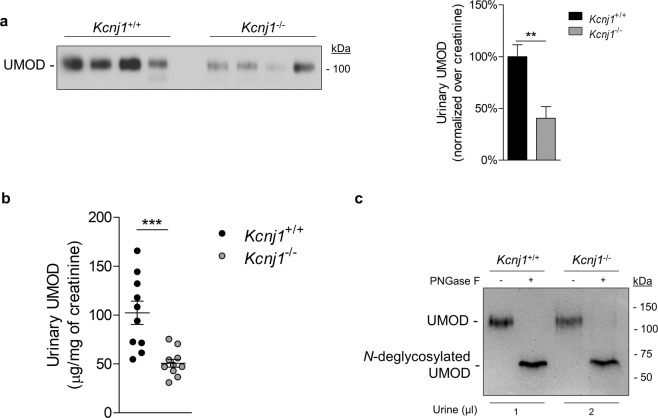
*Kcnj1*^−/−^ mice show reduced urinary uromodulin. (**a**) Representative Western blot of urinary uromodulin (UMOD) in 12 weeks *Kcnj1*^+/+^ and *Kcnj1*^−/−^ mice. Urinary creatinine was used as a loading control. (**b**) Enzyme-linked immunosorbent assay (ELISA) of urinary uromodulin in 12 weeks *Kcnj1*^+/+^ and *Kcnj1*^−/−^ mice. Uromodulin concentration was normalized on urinary creatinine. (**c**) Western blot for uromodulin (UMOD) in control and deglycosylated (PNGase F treatment) urine samples from *Kcnj1*^+/+^ and *Kcnj1*^−/−^ mice. Samples were denatured by heating and treated according to the manufacturer’s instructions. Full length blot images can be found in Supplementary Figure S2.

Immunoblotting experiments showed a ~60% reduction of urinary uromodulin levels (normalized for urinary creatinine levels to account for differences in urine concentration), in *Kcnj1*^−/−^ mice compared to control mice (41 ± 11%, n = 6; p < 0.001) (Fig. 2a). The decreased urinary excretion was confirmed by ELISA (51 ± 4.0 µg/mg creat. vs 102 ± 12 µg/mg creat. in *Kcnj1*^+/+^, n = 10/11, *P* < 0.001) (Fig. 2b). Urinary uromodulin excreted in *Kcnj1*^−/−^ mice retained similar *N*-glycosylation pattern compared to wild-type as PNGase F treatment led to a ~30 kDa shift compared to untreated samples (Fig. 2c).

These data indicate that the constitutive deletion of ROMK is reflected by an apical and cytosolic accumulation of uromodulin in the TAL cells, paralleled by a lower urine excretion of the protein.

### Processing and excretion of uromodulin in *Kcnj1*^fl/fl^*Pax8*^Cre/+^ mice

**Figure 3:**
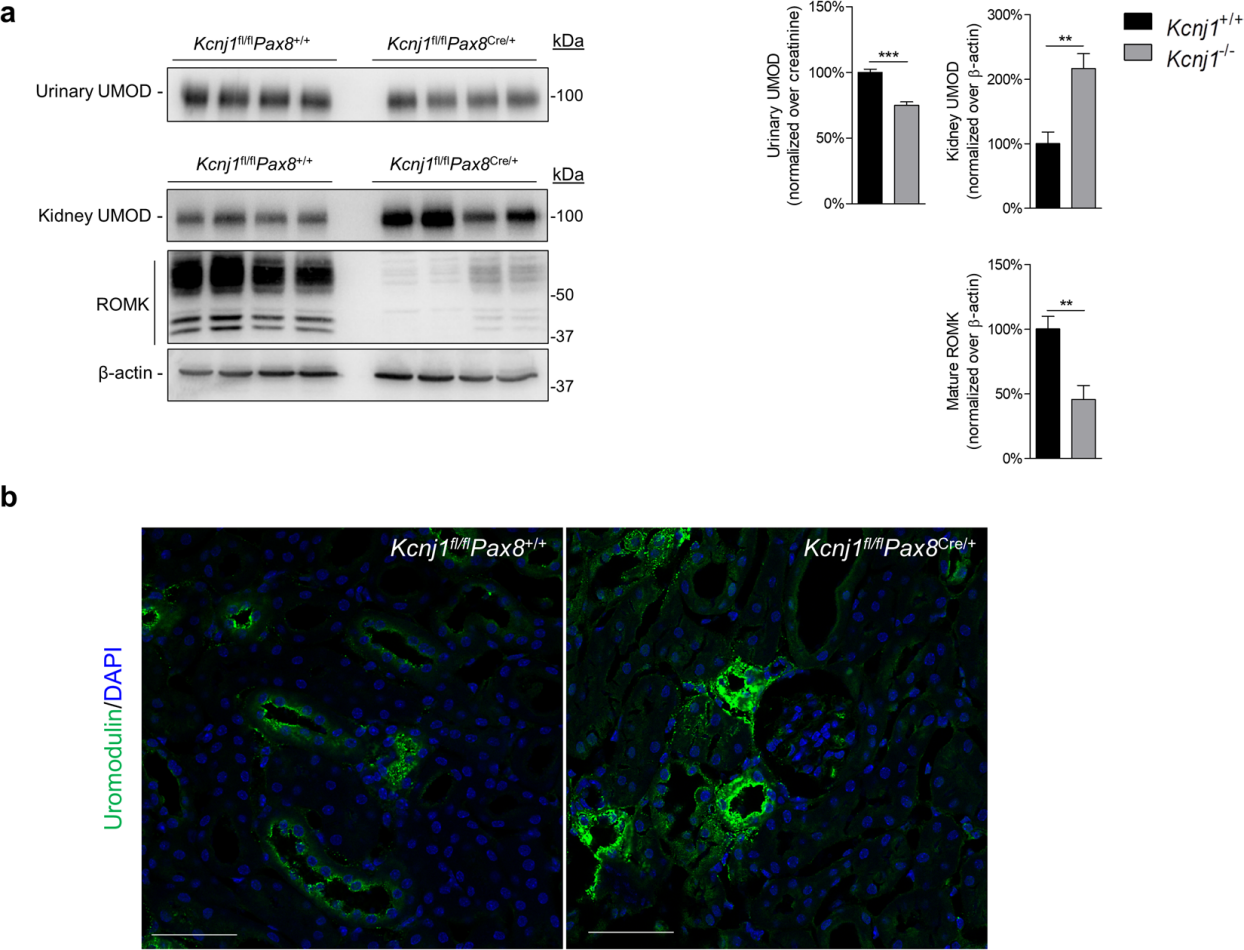
*Kcnj1*^fl/fl^*Pax8*^*Cre*/+^ mice show reduced urinary uromodulin with accumulation in the kidney. (**a**) Representative Western blot analysis for uromodulin (UMOD) and ROMK in urine and whole kidney lysates of *Kcnj1*^fl/fl^*Pax8*^+/+^and *Kcnj1*^fl/fl^*Pax8*^Cre/+^ mice after doxycycline induction. β-actin was used as a loading control. (**b**) Immunofluorescence staining for uromodulin of *Kcnj1*^fl/fl^*Pax8*^+/+^ and *Kcnj1*^fl/fl^*Pax8*^Cre/+^ kidney cryosections (scale bar: 50 µm). Full length blot images can be found in Supplementary Figure S2.

To confirm that the cellular accumulation and reduced urinary excretion of uromodulin is a direct consequence of *Kcnj1* deletion, and not secondary to systemic effects of chronic/global ROMK deletion (constitutive KO), we investigated uromodulin processing in an inducible, kidney-specific *Kcnj1* KO mouse model. This line uses the mouse *Pax8* promoter to drive the expression of a reverse tetracycline-dependent transactivator to obtain an inducible Cre expression in all kidney tubule segments^24^. The Pax8rtTA/LC-1 mice were crossed to transgenic mice harbouring floxed *Kcnj1* (Penton *et al*., MS under revision) allowing to delete *Kcnj1* after treatment with doxycycline. Compared to *Kcnj1*^fl/fl^*Pax8*^+/+^ littermates, *Kcnj1*^fl/fl^*Pax8*^Cre/+^ mice showed a significant reduction in urinary uromodulin (75 ± 3%, n = 4, *P* < 0.001), and a concomitant accumulation of uromodulin in the kidney (216 ± 47%, n = 4, *P* < 0.01) (Fig. 3a) where it is located at the apical pole of the TAL cells (Fig. 3b). These data confirm the direct link between ROMK and uromodulin excretion and processing in the TAL.

### ROMK inhibition in primary mouse TAL cells affects uromodulin processing

**Figure 4:**
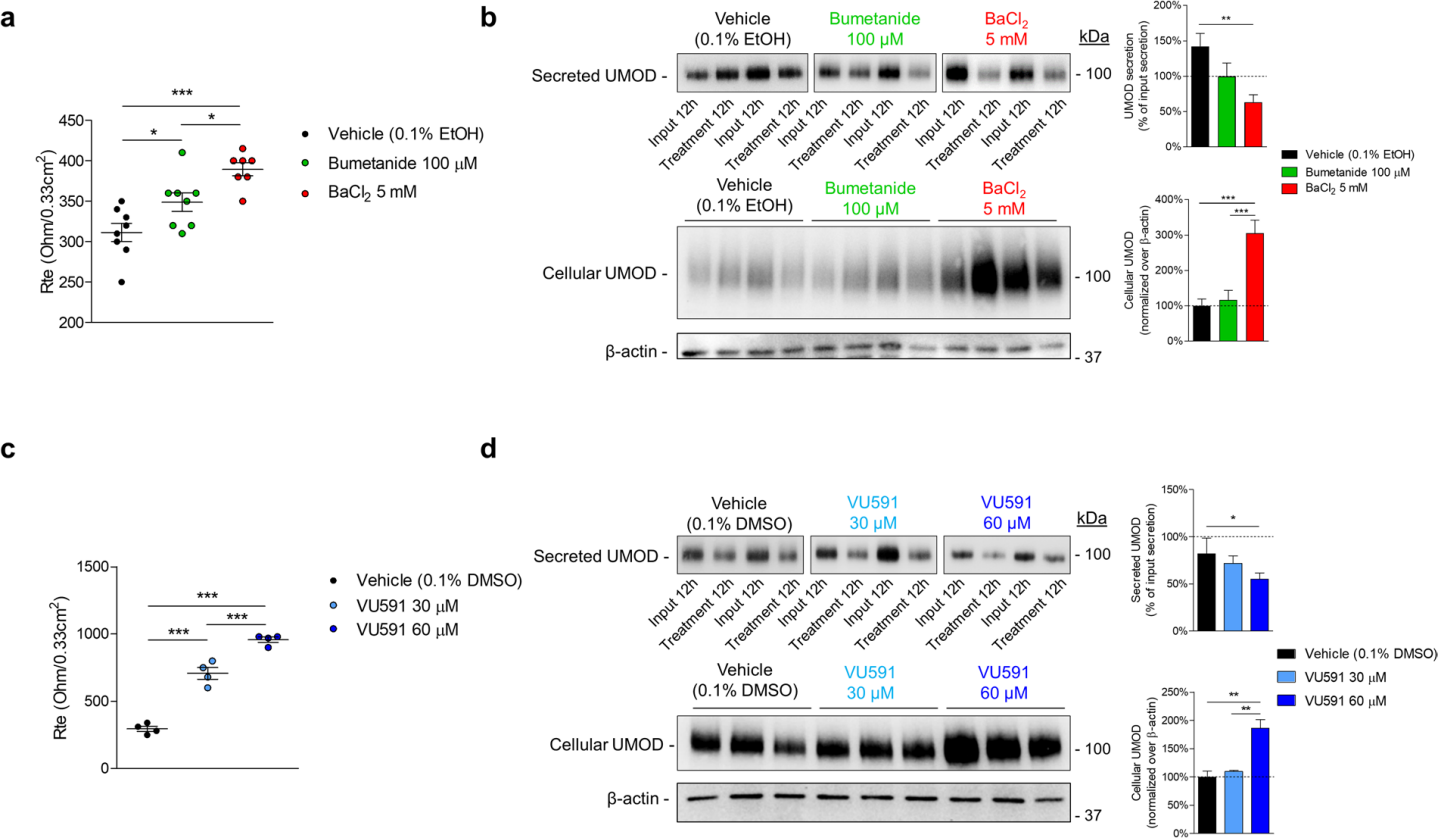
Effects of pharmacological ROMK inhibition on uromodulin processing. (**a**) Transepithelial resistance (R_te_) of primary cultures obtained from microdissected TALs (mTAL) of wild-type mice after 12 hours treatment with 100 µM bumetanide (NKCC2 blocker) or 5 mM BaCl_2_ (K^+^ channels blocker). (**b**) Representative Western blot of secreted and cellular uromodulin (UMOD) in mTAL cells. Apical medium was collected 12 hours before and after treatment with bumetanide or BaCl_2_ (as in panel a). (**c**) Transepithelial resistance (R_te_) of mTAL cells 20 minutes after treatment with either 30 µM or 60 µM VU591 (ROMK inhibitor). (**d**) Representative Western blot of secreted and cellular uromodulin in mTAL cells. Apical medium was collected 12 hours before and after treatment with VU591. Full length blot images can be found in Supplementary Figure S2.

We next used a well-established system of primary mTAL cells obtained from TAL segments isolated from mouse kidneys, in order to directly assess the effect of apical transport systems on endogenous uromodulin processing. The mTAL cells cultured on permeable filters express NKCC2 and ROMK at the apical membrane and generate a lumen-positive transepithelial potential that is abolished by apical bumetanide. Critically, the mTAL cells express endogenous uromodulin, which is processed and secreted in the apical medium similar to the situation observed *in vivo*^25^. Differentiated monolayers of mTAL cells were exposed to non-toxic concentrations of bumetanide (a specific inhibitor of NKCC2) and BaCl_2_ (a blocker of K^+^ channels). Exposure of the apical side of primary TAL monolayers to bumetanide (100 µM) or BaCl_2_ (5 mM) resulted in a robust and sustained inhibition of V_te_ (Suppl. Fig. S1a). Measurement of transepithelial resistance (R_te_) of mTAL monolayers after 12 h exposure to bumetanide (349 ± 11 Ω/0.33 cm^2^, n = 7, *P* < 0.05) and BaCl_2_ (389 ± 8 Ω/0.33 cm^2^, n = 7, *P* < 0.001) revealed a significant increase in R_te_ compared to 0.1% EtOH vehicle (311 ± 11 Ω/0.33 cm^2^, n = 8), indicating an effective blocking of the transport (Fig. 4a). Uromodulin levels in the apical medium were first measured after a 12 h input in order to assess baseline uromodulin excretion rates, and then after 12 h exposure to apical BaCl_2_, bumetanide or in control conditions. Compared to baseline levels, a strong decrease of apical uromodulin excretion was observed in the presence of apical BaCl_2_ (BaCl_2_: 63 ± 11% of input, n = 8, *P* < 0.01) but not with bumetanide (100 ± 19%, n = 8) or 0.1% EtOH vehicle (142 ± 18% of input, n = 8). In parallel, the level of cellular uromodulin was largely increased after BaCl_2_ exposure (305 ± 37% of control, n = 8, *P* < 0.001), whereas it remained unchanged after exposure to bumetanide (116 ± 27% of control, n = 8, *P* = 0.83) (Fig. 4b).

Since BaCl_2_ is a nonspecific K^+^ channel blocker, we exposed mTAL cells to VU591, a selective inhibitor of ROMK that is totally effective at 30 μM in rats^26^. Apical Treatment with VU591 led to a transient decrease in V_te_ (Suppl. Fig. S1b) and a dosage-dependent increase in R_te_ (0.1% DMSO vehicle: 295 ± 19 Ω/0.33 cm^2^, n = 4; 30 μM VU591: 707 ± 44 Ω/0.33 cm^2^, n = 4, *P* < 0.001; 60 μM VU591: 957 ± 19 Ω/0.33 cm^2^, n = 4, *P* < 0.001) (Fig. 4c). This treatment led to a dosage-dependent reduction in the 12 h input-paired excretion of uromodulin to the apical side (0.1% DMSO vehicle: 82 ± 17%, n = 4; 30 μM VU591: 72 ± 8%, n = 4, *P* > 0.05; 60 μM VU591: 55 ± 7%, n = 4, *P* < 0.05) and at the 60 μM dose, an increase of cellular uromodulin (186 ± 15%, n = 3, *P* < 0.01) (Fig. 4d). These changes occurred in absence of any significant effect on cell viability (MTT assay: 95 ± 13% of vehicle, n = 3).

### *Kcnj1* deletion in mTAL cells leads to reduced uromodulin excretion

**Figure 5:**
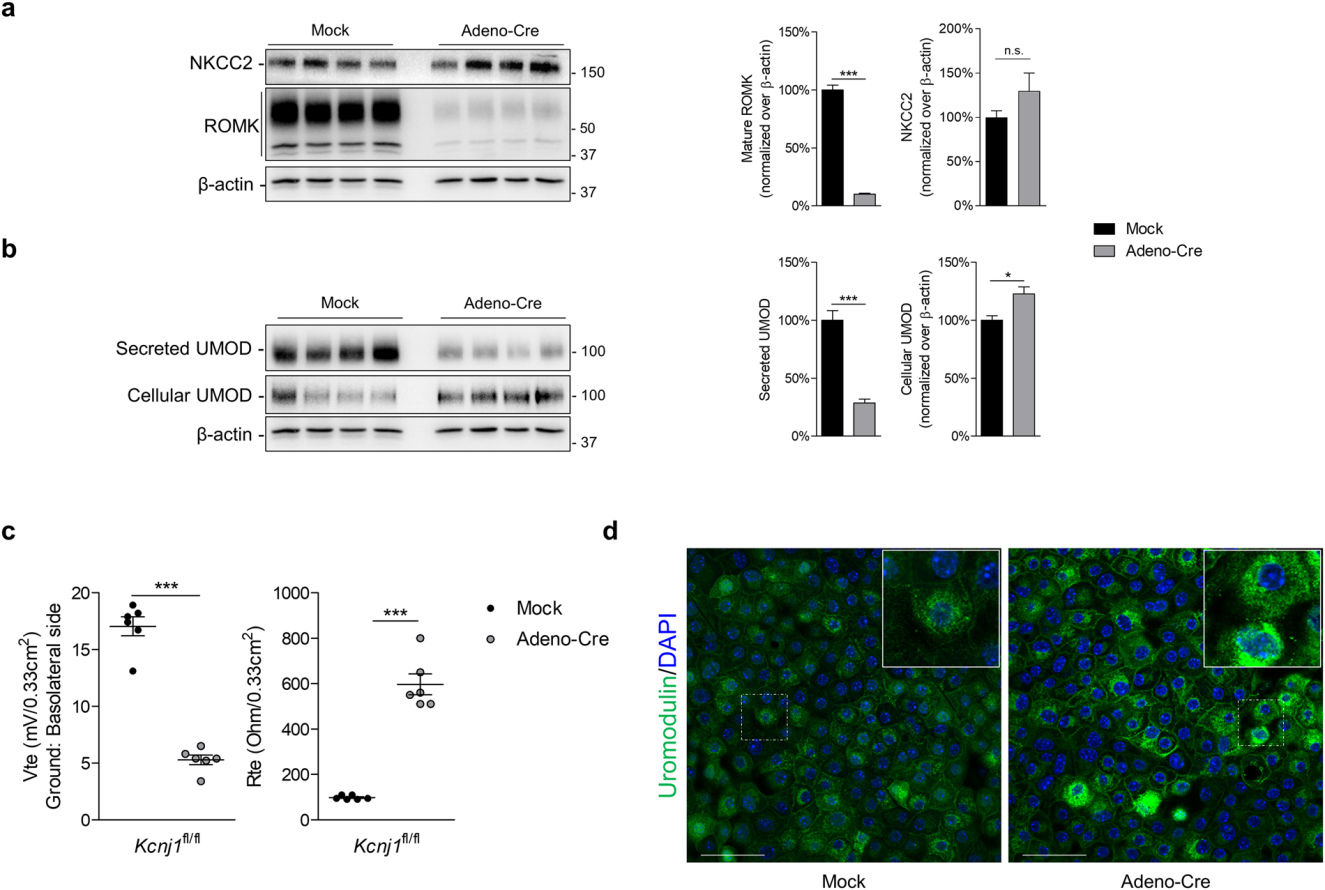
AdenoCre-mediated deletion of *Kcnj1* in primary mouse TAL cells. (**a**) Representative Western blot analysis for NKCC2 and ROMK in primary cultured (mTAL) cells obtained from microdissected TAL segments of *Kcnj1*^fl/fl^ kidneys. Cells were transduced with either an empty adenoviral vector (Mock) or an individual expressing Cre-recombinase (Adeno-Cre). β-actin was used as a loading control. (**b**) Representative Western blot analysis of secreted and cellular uromodulin (UMOD) from *Kcnj1*^fl/fl^ mTAL cells treated with Mock or Adeno-Cre. (**c**) Transepithelial resistance (R_te_) of *Kcnj1*^fl/fl^ mTAL cells 5 days after Mock or Adeno-Cre transduction. (**d**) Immunofluorescence for uromodulin in *Kcnj1*^fl/fl^ mTAL cells after Mock or Adeno-Cre transduction. Full length blot images can be found in Supplementary Figure S2.

We next used mTAL cells to directly test the effect of deleting ROMK on uromodulin processing. To that end, we deleted ROMK in mTAL cells obtained from *Kcnj1*^fl/fl^ mice and transfected with an adenoviral vector expressing either Cre recombinase fused with GFP (AdenoCre) or GFP alone (Mock)^27^. As expected, transfection of mTAL cells with AdenoCre resulted in a specific >90% decrease of ROMK protein expression (10 ± 4%, n = 4, *P* < 0.001) (Fig. 5a), that was reflected by a strong decrease in transepithelial lumen-positive V_te_ and an increase in R_te_ (Fig. 5c). The deletion of ROMK induced a 70% decrease in the apical excretion of uromodulin (28.5 ± 3.4% compared to mock, n = 4, *P* < 0.001) that was paralleled by a significant cellular accumulation as assessed by Western blotting on cell lysate (123 ± 6% compared to mock, n = 4, *P* < 0.05) and immunofluorescent staining on mTAL monolayers (Fig. 5b,d). These data confirm the cell-autonomous link between ROMK activity, transepithelial resistance/voltage and handling of uromodulin in TAL cells.

### Uromodulin excretion is decreased in Bartter-type 2 patients

**Figure 6:**
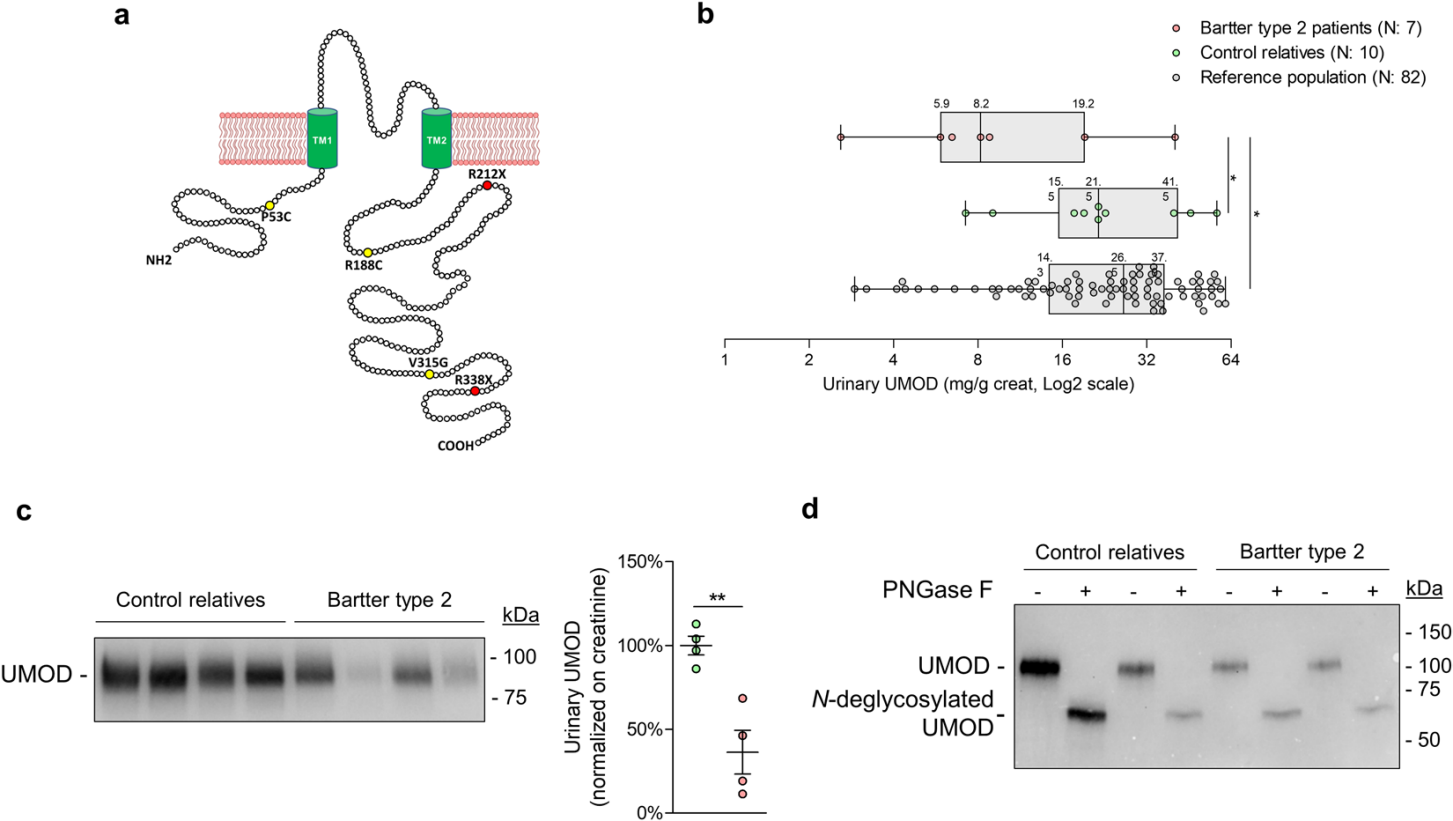
Urinary uromodulin levels in Bartter type 2 patients. (**a**) Amino acid composition of ROMK, with mutations found in Bartter type 2 patients (yellow, missense; red, nonsense). TM: Transmembrane domain. (**b**) Box and whiskers plot of urinary uromodulin (UMOD) levels of Bartter type 2 patients, control relatives and a healthy reference population (age ≤ 20, eGFR ≥ 80). Median, 25^th^ and 75^th^ percentiles are indicated on the respective plot. (**c**) Representative Western blot analysis of UMOD in urine samples from Bartter type 2 patients and control individuals. Samples were loaded according to urinary creatinine. (**d**) Western blot analysis of UMOD in control and deglycosylated (PNGase F treatment) urine samples from Bartter type 2 patients and control relatives. Samples were denatured by heating and treated according to the manufacturer’s instructions. Full length blot images can be found in Supplementary Figure S2.

The translational relevance of the link between ROMK function and uromodulin excretion was tested in a cohort of patients with antenatal Bartter syndrome type 2 due to *KCNJ1* mutations (Fig. 6a; Suppl. Table S1). When compared to the distribution of urinary uromodulin concentrations (as assessed by ELISA^28^ and normalized to creatinine) in a GFR-matched healthy reference population (n = 82), uromodulin concentration was below the 25^th^ percentile of the reference population in 5/7 patients. In contrast, age and gender-matched control individuals from the same families (n = 10) had urinary uromodulin levels comparable to the reference population. The urinary uromodulin level was significantly lower in Bartter type 2 patients when compared to related control individuals (13.1 ± 5.0 vs. 26.2 ± 5.1 µg/mg creat, respectively, *P* < 0.05) and to the reference population (27.6 ± 1.8 µg/mg creat, *P* < 0.05) (Fig. 6b; Suppl. Table S2). Western blot analysis of urine samples also showed a ~70% reduction in the level of uromodulin normalized to creatinine in Bartter 2 patients compared to the related controls (36 ± 13%, n = 4, *P* < 0.01) with no apparent shift in molecular weight in control and deglycosylated conditions (Fig. 6c).

## Discussion

Despite the identification of important roles of uromodulin in physiology and disease over the last decade, the regulatory mechanisms involved in its production, processing and release into the urine remain poorly characterized. Based on evidence obtained from cellular studies, mouse models and human samples, we establish for the first time a link between the function of the K^+^ channel ROMK and the urinary excretion of uromodulin. We show that constitutive or induced genetic deletion of ROMK or its pharmacologic blockage result in defective apical excretion of uromodulin. The levels of uromodulin in urine are also significantly decreased in patients harbouring loss-of-function mutations in *KCNJ1* responsible for Bartter syndrome type 2, compared to levels observed in age-matched controls from the same family or from a reference population.

Uromodulin is an abundant protein essentially produced in the epithelial cells lining the TAL, where it is apically targeted and released in the urine following proteolytic cleavage^1^. The TAL cells play a major role in the reabsorption of NaCl and divalent cations, and in the generation of the osmotic gradient allowing to concentrate urine. The transport processes operating in the TAL are somehow linked, as evidenced by the phenotype overlap observed in the various types of Bartter syndrome^29^. In fact, early studies suggested that the levels of uromodulin excretion may be lower in at least some patients with clinically diagnosed (but not genetically proven) Bartter syndrome^30,31^. The hypothesis of a link between uromodulin excretion and ROMK was substantiated by a meta-GWAS followed by a candidate gene approach for urinary uromodulin level in 10,884 individuals of European ancestry^15^. Among 24 candidate genes expressed and known to be involved in transport processes in the TAL, the most significant SNP associated with the urinary level of uromodulin was identified in *KCNJ1* coding for ROMK^15^. Also, uromodulin levels were shown to correlate with tubular handling of electrolytes in population-based studies^21,22^.

We provide multi-level evidence supporting a direct link between the expression and activity of ROMK and the processing and excretion of uromodulin by TAL cells. *In vivo* studies in constitutive and inducible *Kcnj1* KO mouse models demonstrate that deletion of ROMK leads to a marked defect in uromodulin processing in the TAL, as indicated by intracellular accumulation and lower urine excretion. The similar effects observed in the two models indicate that the influence of ROMK on uromodulin processing is independent of any long-term effect secondary to its deletion. We substantiated the link between ROMK and uromodulin using mTAL cells, which endogenously express uromodulin^25^. Pharmacologic blockade of ROMK using non-selective BaCl_2_ or selective VU591, which led to the expected increase of transepithelial resistance (R_te_), was associated with a marked cellular accumulation and a decrease in the apical excretion of uromodulin. The effect was specifically due to ROMK, since NKCC2 inhibition by bumetanide did not significantly reduce uromodulin excretion.

Because uromodulin excretion was significantly reduced after pharmacological inhibition and genetic deletion of ROMK and in patients harbouring *KCNJ1* mutations, we can assume that ROMK channel function and not the protein expression *per se* modulates uromodulin excretion. Inwardly rectifying potassium (K_ir_) channels are important regulators of resting membrane potential and ROMK is critical for maintaining the lumen-positive transepithelial voltage in the TAL^32^. In some cell types, membrane potential has been shown to modulate the trafficking of membrane proteins^33^. It would be interesting to test whether such membrane potential-dependent trafficking mechanism could contribute to the observed alterations in uromodulin excretion. Due to its abundant sugar moieties, uromodulin presents as a negatively charged protein at physiological pH and is associated with lipid rafts^1^. Lipid rafts themselves are rich in gangliosides and negatively charged and have been shown to act as electric field sensors and to polarize when exposed to an electric current^34^. The physiological TAL lumen-positive voltage might thus contribute to a preferential apical raft (and uromodulin) distribution that is disrupted in ROMK deficient cells.

The Bartter type 2 patients investigated here were taking numerous medications at time of sampling, including NaCl and KCl supplements, calcium and vitamin D, and indomethacin. The potential effect of these medications (individual or in combination) on the processing and urinary levels of uromodulin has not been systematically tested. It should also be pointed that the defect in uromodulin processing might contribute to the Bartter syndrome phenotype. The expression of uromodulin modulates the activity of NKCC2 and ROMK in the TAL^7,19^, and its deletion in mouse leads to a discrete salt-losing phenotype^35^. We recently showed that defective processing of uromodulin (due to hepsin mutation) is causing TAL dysfunction and salt wasting^36^. All these effects could contribute to the tubular dysfunction typically observed in patients with Bartter syndrome.

Altogether, our data demonstrate that deficient ROMK function affects the processing and urinary excretion of uromodulin, supporting the mutual dependence of critical processes taking place in the TAL.

## Methods

### Animal models, biochemical analyses and tissue collection

*Kcnj1*^−/−^ mice^37^ and wild-type (*Kcnj1*^*+*/+^) littermates were housed in a light- and temperature-controlled environment with *ad libitum* access to tap water and standard chow (Diet AO3, SAFE; 25/18 GR Mucedola Srl, Settimo Milanese, Italy). Urine samples were collected using individual metabolic cages (UNO Roestvastaal BV, Zevenaar, The Netherlands) after appropriate training. Blood was collected by venous puncture or cardiac puncture at time of sacrifice and centrifuged at 2000 g for 15 minutes at 4 °C in heparin-coated tubes (Sarstedt AG, Nürnbrecht, Germany) in order to separate plasma from cells. Creatinine, electrolytes and urea were measured on a Synchron CX5 analyser (Beckman Coulter, Fullerton, CA, USA), according to the manufacturer’s instructions. Urine and plasma osmolality were measured on a multi-sample osmometer (Advanced Instruments Model 2020, Norwood, MA, USA). Urinary concentration of uromodulin in mouse urine was assessed by means of a commercial ELISA kit (ab245726, Abcam, Cambridge, UK). Following the collection of urine and plasma samples, mice were sacrificed either by decapitation or by cervical dislocation following anaesthesia with isoflurane (Minrad International Inc., Orchard Park, NY, USA) for the collection of the kidneys. One kidney was immediately homogenized for protein or RNA extraction, while the other was further processed for histological analyses. *Kcnj1*^fl/fl^ mice were generated by Ozgene (Perth, Australia) as described elsewhere (Penton *et al*., MS under revision) and were used to obtain kidney tissue for primary mTAL cell culture. Moreover, kidney lysates, frozen tissues and urine samples were obtained from tetracycline-inducible kidney-specific *Kcnj1* KO mice that had been generated by crossing the *Kcnj1*^fl/fl^ mice with Pax8-rtTA-cre mice^24^. All of the experiments were performed in accordance with the ethical guidelines at University of Zurich (Zurich, Switzerland) and the legislation of animal care and experimentation of Canton Zurich, Switzerland. The experimental protocols were approved by the appropriate licensing committee (Kanton Zürich Gesundheitsdirektion Veterinäramt; protocol ZH049/17) at the University of Zurich.

### Protein samples preparation and immunoblotting

Proteins were extracted from grinded tissue using ice-cold RIPA buffer (Sigma-Aldrich, St.-Louis, MO, USA) with the addition of protease and phosphatase inhibitors (Roche, Basel, Switzerland), followed by a brief sonication and a centrifugation for 15 min at 1000 g and 4 °C to remove debris. For the membrane-cytosolic separation, mouse kidneys were solubilized in sucrose buffer (1 mM EDTA, 20 mM Imidazole and 250 mM Sucrose, pH 7.2) and pellets enriched in large plasma membranes (“membrane fraction”) (16,000 g, 1 h, 4 °C) and in cytoplasmic proteins and cytoplasmic vesicles (“cytosolic fraction”) (200,000 g, 2 h, 4 °C) were sequentially collected. Protein concentrations in kidney/mTAL lysates were determined using the bicinchoninic acid (BCA) protein assay kit (Thermo Fischer Scientific, Waltham, MA, USA), while urine was loaded according to creatinine concentration. For semi-quantitative Western blot analysis, urine samples were denatured by boiling but not reduced. For qualitative Western blot analysis, all urine samples were denatured by boiling and reduced using DTT. When indicated, these samples were N-deglycosylated using PNGase F (P70404L, New England Biolabs, Ipswich, MA, USA) according to the manufacturer’s instructions. Briefly, urine samples were prepared by adding the denaturating buffer and heating for 10 minutes at 100 °C. The final reaction was prepared by adding 10% NP-40, G7 Reaction Buffer and PNGase F (H_2_O for negative controls) to the denatured samples, which were then incubated for 1 hour at 37 °C. Kidney samples were thawed on ice, normalized for protein levels, diluted in Laemmli sample buffer (Bio-Rad Laboratories Inc., Hercules, CA, USA), separated on a 7.5–10% SDS-PAGE gel and blotted onto methanol-activated PVDF membranes. Membranes were blocked for 30 min in 5% w/v non-fat dry milk solution at room temperature, followed by overnight incubation at 4 °C with primary antibodies. Thereafter, blots were washed and incubated with peroxidase-conjugated secondary antibodies, washed again and visualized by Immun-Star™ enhanced chemiluminescence (Bio-Rad). Immunoblots were quantified by densitometry using ImageJ^38^.

### Histological analysis and immunostaining

Kidneys were fixed overnight at 4 °C in 4% formaldehyde (Sigma-Aldrich), dehydrated and subsequently embedded in paraffin. Paraffin blocks were cut into 5 µm-thick sections, deparaffinized in xylene and re-hydrated in decreasing ethanol concentrations.

Haematoxylin and Eosin (Sigma-Aldrich) staining was performed according to standard protocols and the staining was evaluated by use of a Zeiss MIRAX MIDI slide scanner (Carl Zeiss, Oberkochen, Germany). Immunostaining was performed on paraffin sections and/or 5 μm thick cryosections obtained from *Kcnj1*^−/−^ and *Kcnj1*^+/+^ kidneys. Sections were deparaffinized in xylene and rehydrated using decreasing ethanol concentrations. Heat mediated antigen retrieval was performed using 10 mM citrate buffer (pH 6.0) for 10 min at 98 °C in a Histos Pro Rapid Microwave Histoprocessor (Milestone Inc., Shelton, CT). The sections were blocked for 30 min in PBS containing 10% normal goat serum (CL1200, Cedarlane Laboratories, Burlington, Canada) at room temperature and incubated with the primary antibody in a humidified chamber for either 2 hours at RT or overnight at 4 °C. The sections were then incubated with secondary antibodies in PBS containing 0.5% BSA for 2 hours at RT, washed and mounted using Prolong Gold Anti-fade reagent with DAPI (P36931, Thermo Fisher Scientific).

### Isolation, culture and treatment of primary mouse TAL cells

Primary cell cultures of mouse TAL (mTAL) cells were prepared from wild-type C57/BL6 (Charles River, Suzbach, Germany) mice and *Kcnj1*^fl/fl^ mice as previously described^25^. Briefly, TALs were isolated under a light microscope on the basis of morphology characteristics and cultured on permeable filter supports (Transwell-COL, pore size 0.4 µm, Corning Costar, USA) containing a DMEM:F12-based medium for 7–10 days in a humidified chamber at 37 °C and 5% CO_2_ until confluent monolayers were formed. For pharmacological inhibition, primary mTAL cultures were treated at the apical side of the filter with 100 µM bumetanide (B3023, Sigma-Aldrich) or 5 mM BaCl_2_ (342920, Sigma-Aldrich) for 12 hours, and 30–60 µM VU591 (SML0077, Sigma-Aldrich) followed by 2 days of serum-free conditions. Before and after each experiment the apical medium was collected for further analysis. For expression studies, adenovirus constructs used include eGFP under control of a CMV promoter (#1060, Vector Biolabs, Malvern, PA, USA) or an individual carrying also Cre-recombinase (#1700, Vector Biolabs). The transduction protocol was performed as previously described^27^. Briefly, the cells were plated onto filters and transduction was performed 24 h after plating, when cells reached approximately 70–80% of confluence. The cells were subsequently incubated for overnight at 37 °C with culture medium containing the virus at the appropriate concentration (0.2125 × 10^9^ PFU/mL). Culture medium was changed every other day, and the cells were collected for analysis after 5 days. Cultures were subjected to transepithelial resistance (R_te_) measurements using an EVOM-G potentiometer (World Precision Instruments, Sarasota, FL, USA) and Endohm 6 electrodes (WPI). At the end of the experiments, cells were either lysed for RNA/protein collection or fixed for immunofluorescence staining. Thiazolyl Blue Tetrazolium Bromide (MTT) assay was performed according to manufacturer instructions. Briefly, cells were incubated with 5 mg/mL MTT (Sigma-Aldrich) for 2 hours at 37 °C. MTT formazan crystals were then solubilised with DMSO and the optical density was measured at 565 nm using an Infinite M Plex microplate reader (Tecan, Männedorf, Switzerland).

### Immunofluorescence staining of mTAL cells

Confluent monolayers of primary cultured mTAL cells were fixed for 20 min on ice with 4% (w/v) formaldehyde (Sigma-Aldrich), quenched for 10 min at room temperature (RT) and permeabilised for 15 min at 37 °C in permeabilisation solution (PS: 0.7% gelatin (#G7041-100G, Sigma-Aldrich) and 0.016% saponin (#47036-50GF, Sigma-Aldrich) in H_2_O). A primary antibody detecting uromodulin was diluted in PS and incubated for 1 h at 37 °C. After four washing steps in PS, monolayers were incubated for 30 min at 37 °C in PS with a donkey anti-sheep Alexa488 (#A-11015, Thermo Fisher Scientific) secondary antibody. Next the cells were washed 3-times in PBS containing 0.1% Triton-X (#T9284, Sigma-Aldrich), post-fixated for 15 min at RT using 4% formaldehyde in PBS containing DAPI (Life technologies, #D1306), and rinsed once with PBS. The filters were cut from the holder and mounted in Prolong Gold Anti-fade reagent (Thermo Fisher Scientific). Sections and cells were viewed using a LSM510Meta Confocal microscope (Carl Zeiss, Oberkochen, Germany), with an ×63/1.4 Plan- Apochromat oil-immersion objective; x40/NA 1.25 oil immersion objective and an x20/ NA 0.7 multi immersion objective.

### RNA isolation, reverse transcription and quantitative PCR

Analysis of gene expression levels in mouse kidneys and mTAL cells were performed based on the MIQUE guidelines^39^. In brief, the extraction of total RNA from mTAL cells was performed using RNAqueous-Micro kit (Ambion, Huntingdon, UK). Total RNA was extracted from kidney using Aurum TM Total RNA Fatty and Fibrous Tissue Kit (Bio-Rad, Hercules, CA), following the protocol of the manufacturer. Contamination by genomic DNA was eliminated by DNase I treatment. Reverse transcriptase reaction with iScript TM cDNA Synthesis Kit (Bio Rad) was executed with up to 1 µg of RNA. When needed, PCR products were sizefractionated on 1.5% agarose gel and stained with EZ-VisionR One (AMRESCO, Solon, OH). The variations in mRNA levels of the target genes were established by relative RT-qPCR with a CFX96TM Real-Time PCR Detection System and the iQ™ SYBR Green Supermix (Bio-Rad) for the detection of single PCR product accumulation. 100 nM of sense and anti-sense primers were used in a final volume of 20 µl in iQ™ SYBR Green Supermix (Bio-Rad) to perform RT-qPCR analyses (in duplicate). Primers specific to targets were designed with Beacon Design 2.0 (Premier Biosoft International, Palo Alto, CA). PCR conditions were: 95 °C, 3 min followed by 40 cycles of 15 sec, 95 °C and 30 seconds at 60 °C. The efficiency of each set of primers was determined by dilution curves (Suppl. Table S3). For TAL cell expression studies, *Gapdh* was used as a reference gene. GeNorm version 3.4 was used to characterize the expression stability of the candidate reference genes in kidney. Normalization factor was determined using 6 housekeeping genes (*18 S*, 3*6B4*, *Actb*, *Gapdh*, *Hprt1*, *Ppia*)^40^. The relative changes were determined by the formula: 2^−ΔΔct^ and expressed as percentage relative to wild-type.

### Urinary uromodulin measurements

Patients with established diagnosis of antenatal Bartter syndrome type 2 and age- and gender-matched relatives were recruited (Suppl. Table S1). The reference samples were obtained from participants of the population-based, Swiss Kidney Project on Genes in Hypertension (SKIPOGH) study, and matched for age and eGFR^41^. Recruitment procedures have been previously described^41,42^. All patients and controls gave informed consent. The experimental protocols were approved by the Ethics Committee of the Universities of Bern, Geneva, and Lausanne (Switzerland); the University of Louvain (Brussels, Belgium); and the EURenOmics consortium (FP7, 2007–2013, grant agreement no. 305608). All experiments were performed in accordance with relevant guidelines and regulations.

Urinary uromodulin concentration was measured using a well-established ELISA based on a sheep anti-human uromodulin antibody as the capture antibody, a mouse monoclonal anti-human uromodulin detection antibody, and a goat anti-mouse IgG horseradish peroxidase–conjugated protein (#172-1011, Bio-Rad) as a secondary antibody^28^. For detection, a 250 µg/L O-Phenylenediamine dihydrochloride (OPD) substrate solution was freshly prepared in phosphate-citrate buffer (0.1 M citric acid monohydrate, 0.2 M Na_2_HPO_4_, 0.006 M H_2_O_2_, pH 5.5). Human uromodulin (Millipore, Billerica, MA, USA) was used to establish the standard curve. The ELISA assay has a sensitivity of 2.8 ng/ml and a linearity of 1.0^28^. Urinary creatinine levels were measured using Beckman Coulter Synchron® System Creatinine Assay (Unicell DxC Synchron® clinical System), according to manufacturer instructions. Uromodulin was indexed to creatinine to correct for variations in urine concentration.

### Antibodies

The following primary antibodies were used: sheep anti-uromodulin (K90071C, Meridian Life Science Inc., Cincinnati, OH, USA; 1:500 for WB and 1:300 for IF), mouse anti-uromodulin (CL 1032 A; Cedarlane Laboratories; for ELISA), mouse anti-flotillin-1 (BD 610821, BD Biosciences, Franklin Lakes, NJ, 1:500 for WB), rabbit anti-GAPDH (2118, Cell Signaling Technology, Danvers, MA, USA; 1:500 for WB), mouse anti-β actin (A5441, Sigma-Aldrich; 1:10 000 for WB, rabbit anti-NKCC2 (AB3562P, Merck-Millipore, Burlington, MA, USA; 1:500 for WB and 1:100 for IF), rabbit anti-ROMK (Penton *et al*., MS under revision; 1:1000 for WB and 1:5000 for IF).

### Statistical analysis

All values are expressed as mean ± SEM. Comparisons between groups were performed using Student’s t-test or one-way ANOVA in case of 3 or more groups’ comparisons. In the latter case, Tukey-Kramer’s post hoc analysis was also performed. P values ≤ 0.05 were considered statistically significant.

## Supplementary information

Supplementary Information
