# Supplementary material for: The Urinary Excretion of Uromodulin is Regulated by the Potassium Channel ROMK
Source: Sci Rep. 2019 Dec 20;9:19517. doi: 10.1038/s41598-019-55771-x (PMC6925250; doi:10.1038/s41598-019-55771-x)
Supplement: Supplementary file 1 — Supplementary Information [file 41598_2019_55771_MOESM1_ESM.pdf]

The Urinary Excretion of Uromodulin is Regulated by the Potassium Channel ROMK

Guglielmo Schiano, Bob Glaudemans, Eric Olinger, Nadine Goelz,

Michael Müller, Dominique Loffing-Cueni, Georges Deschenes, Johannes Loffing & Olivier Devuyst

Supplementary Material

Suppl. Figure 1-2
Suppl. Tables 1-3

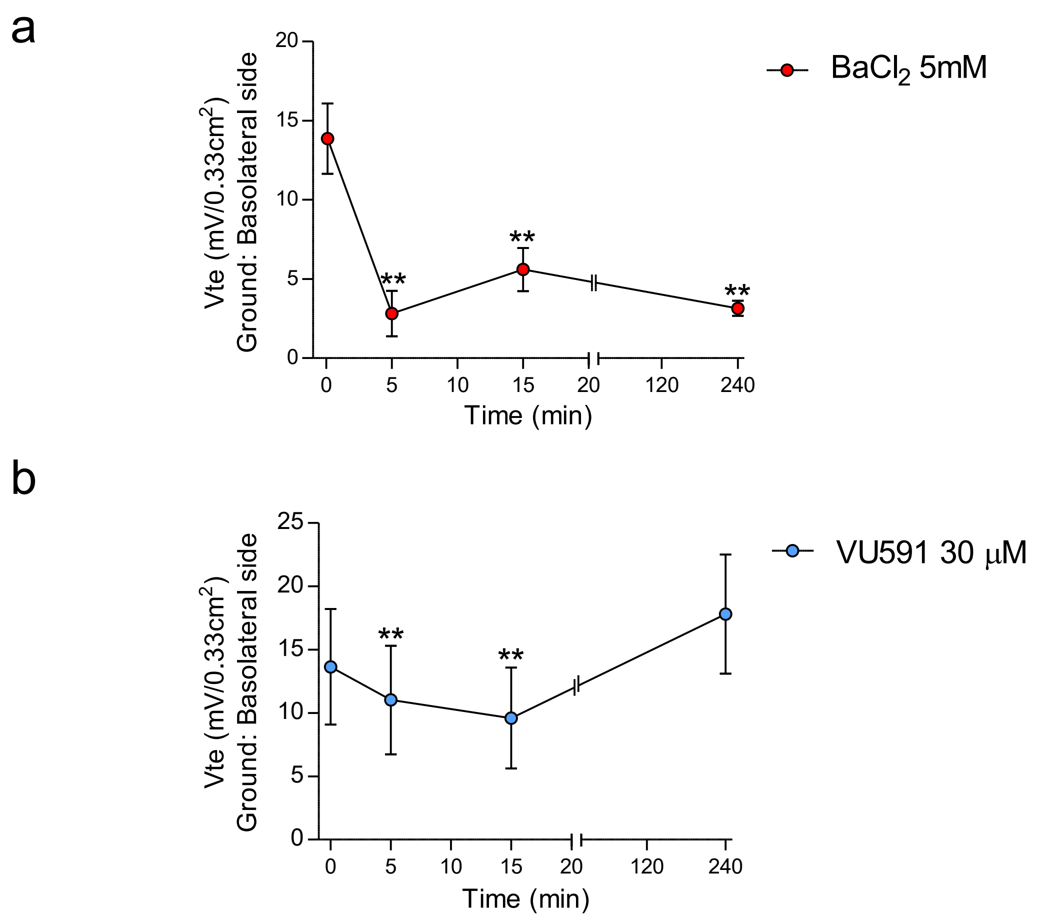

Figure S1. Transepithelial voltage decrease following ROMK inhibition.
Transepithelial voltage (V_{te}) measurements following (**a**) treatment with $BaCl_2$ 5 mM and (**b**) treatment with VU591 30 μM (n=4). Each sample was compared to their respective baseline value using a paired Student's t test. Values are expressed as mean ± SEM. $^{**}P \leq 0.01$

Figure S2. Full Western blot membranes.

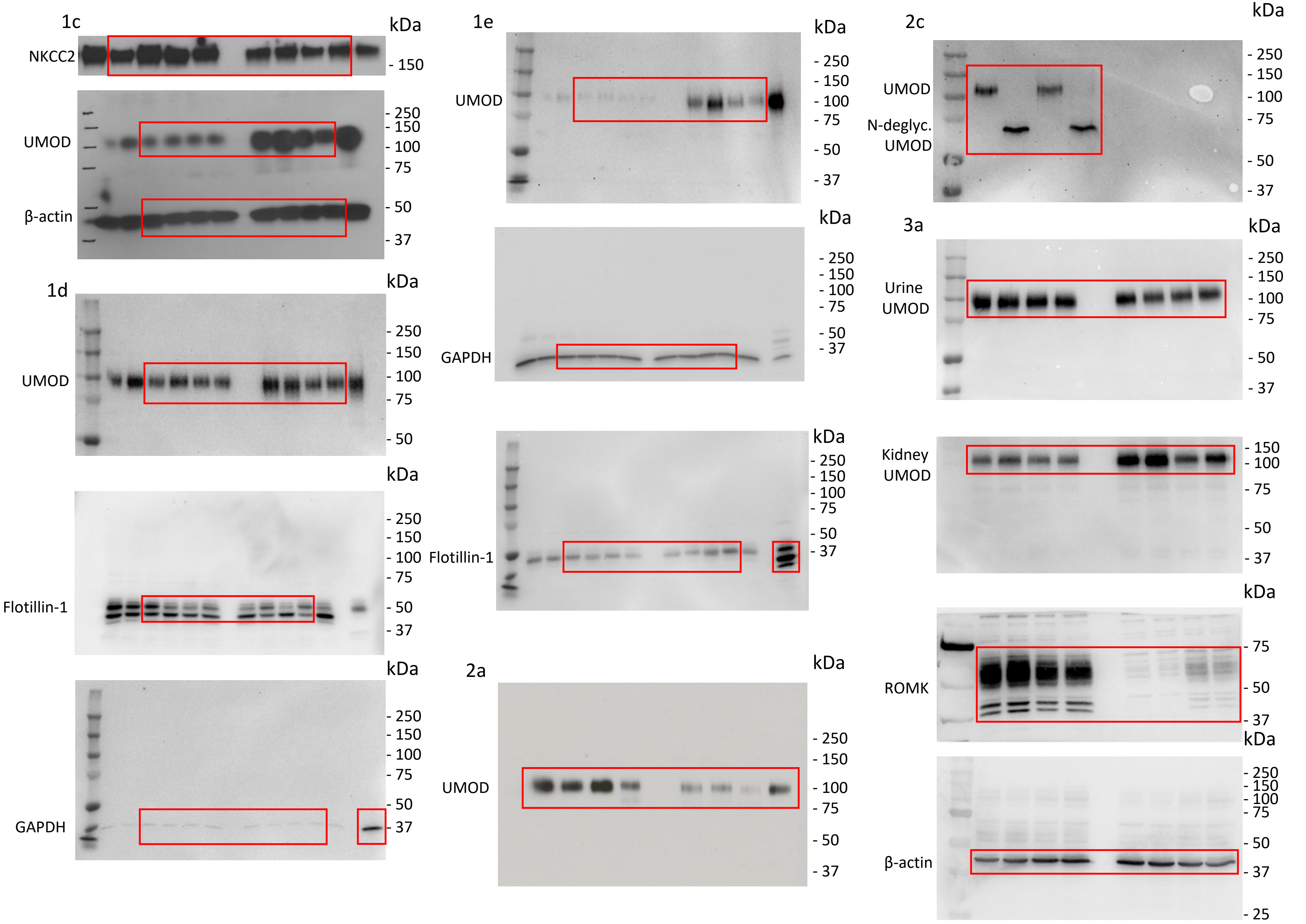

Figure S2 (continued).

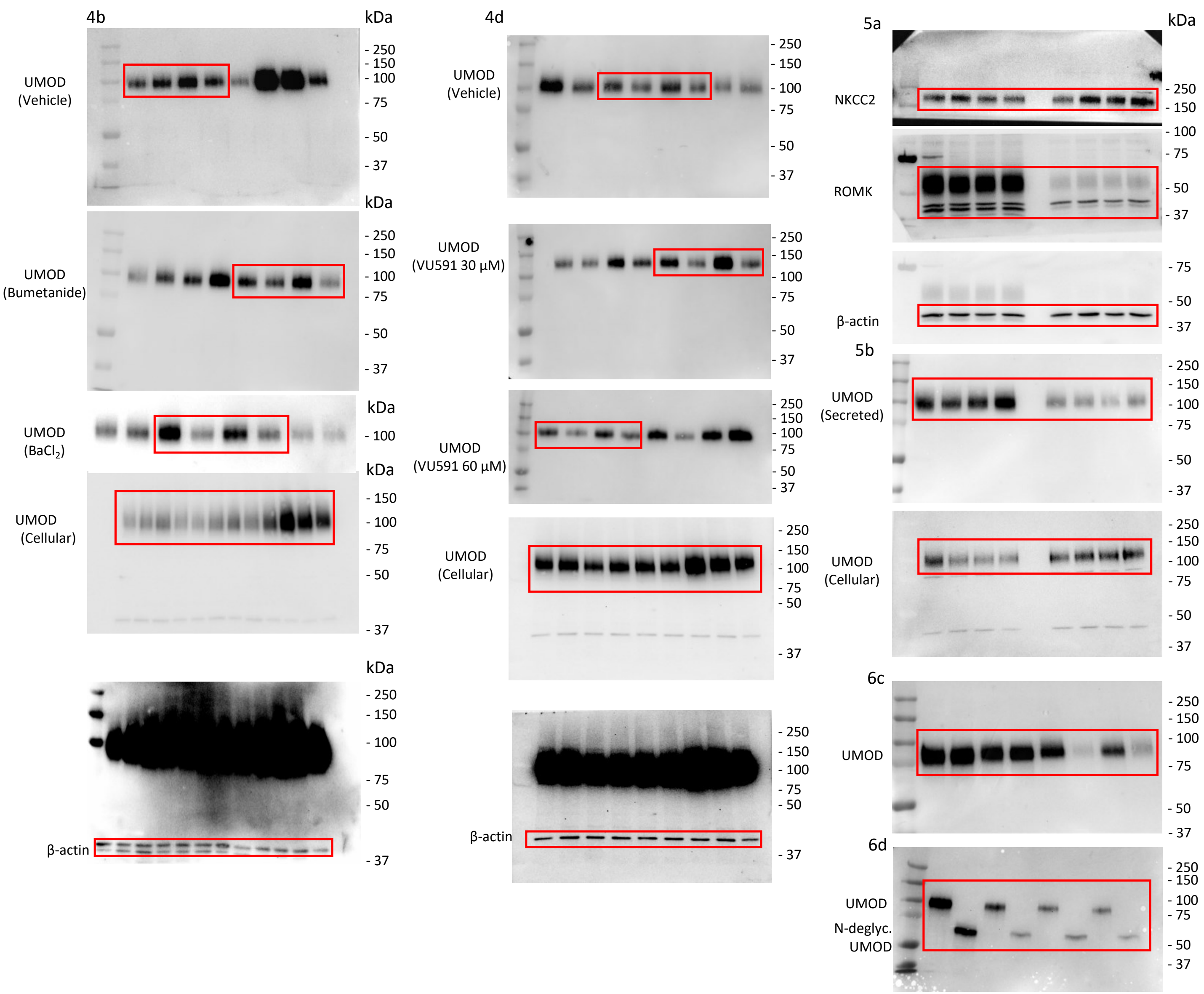

Table S1. *KCNJ1* mutations in patients with Bartter syndrome type 2.

**Gender**	**Genetic status (*KCNJ1*)**
M	c.1012C>T p.Arg338X (homozygous)
F	c.1012C>T p.Arg338X (homozygous)
F	c.634C>T p.Arg212X c.158T>G p.Phe53Cys
M	c.944T>G p.Val315Gly (homozygous)
F	c.1505T>G p.Val315Gly (homozygous)
M	c.552C>T p.Arg188Cys (heterozygous)
F	c.552C>T p.Arg188Cys (heterozygous)

M, male; F, female

Table S2. Characteristics of Bartter type 2 patients, control relatives and reference population.

	N	Gender (M/F)	Mean age (years)	Median urinary UMOD (µg/mg creatinine)	25th – 75th percentile	Mean eGFR (mL/min/1.73 m^2)
Bartter type 2 patients	7	3/4	12.3 ± 2.2	8.17	5.9 – 19.2	90 ± 3
Control relatives	10	6/4	11.5 ± 2.2	21.52	15.5 – 41.5	ND
Reference population (age≤20 y, eGFR≥80)	82	48/34	18.9 ± 0.1	26.46	14.3 – 37.0	122± 1

M, male; F, female; eGFR, estimated glomerular filtration rate; ND, not determined. See Methods for population description.

Table S3. Primers used for real-time RT-PCR analysis.

Gene product	Forward primer (5'-3')	Reverse primer (5'-3')	PCR Product (bp)	Efficiency
18S	GTA ACC CGT TGA ACC CCA TT	CCA TCC AAT CGG TAG TAG CG	151	0.98±0.02
36B4	CTT CAT TGT GGG AGC AGA CA	TTC TCC AGA GCT GGG TTG TT	150	1.02±0.02
Actb	TGC CCA TCT ATG AGG GCT AC	CCC GTT CAG TCA GGA TCT TC	102	1.03 ± 0.04
Aqp1	GCT GTC ATG TAC ATC ATC GCC CAG	AGG TCA TTG CGG CCA AGT GAA T	107	0.99 ± 0.03
Aqp2	TGA GCC TCA AGA AGG GTC TC	TCT CCA GAG CTC TCC GTC TC	142	0.98 ± 0.03
Clcnkb	GGC TAC CAG CAA ACC CTT GT	CAT CAG TGC CCA GGA GTT GT	151	1.01 ± 0.03
Cldn16	TTT GAT GGG ATT CGA ACC TG	CAG TCC AGA CCA AGG AGC A	143	0.98 ± 0.04
Gapdh	TGC ACC ACC AAC TGC TTA GC	GGA TGC AGG GAT GGG GGA GA	176	1.04 ± 0.03
Hprt 1	ACA TTG TGG CCC TCT GTG TG	TTA TGT CCC CCG TTG ACT GA	162	0.99 ± 0.01
Kcnj1	CCG TGT TCA TCA CAG CCT TCT T	CCG TAA CCT ATG GTC ACT TGG G	190	1.03 ± 0.03
Nphs2	GTC TAG CCC ATG TGT CCA AA	CCA CTT TGA TGC CCC AAA TA	162	1.03 ± 0.03
Ppia	CGT CTC CTT CGA GCT GTT TG	CCA CCC TGG CAC ATG AAT C	139	1.02 ± 0.02
Pvalb	GAC GCC ATT CTT CTG GAA AT	ATA CCC CCA CTG CCC TAA AA	136	0.98 ± 0.02
